# Large conductance voltage-and calcium-activated K^+^ (BK) channel in health and disease

**DOI:** 10.3389/fphar.2024.1373507

**Published:** 2024-03-22

**Authors:** Felipe Echeverría, Naileth Gonzalez-Sanabria, Rosangelina Alvarado-Sanchez, Miguel Fernández, Karen Castillo, Ramon Latorre

**Affiliations:** ^1^ Centro Interdisciplinario de Neurociencia de Valparaíso, Instituto de Neurociencia, Facultad de Ciencias, Universidad de Valparaíso, Valparaíso, Chile; ^2^ Centro de Investigación de Estudios Avanzados del Maule, Vicerrectoría de Investigación y Postgrado, Universidad Católica del Maule, Talca, Chile

**Keywords:** BK channel, KCNMA1, channelopathies, physiology, molecular and cellular mechanisms, cellular excitability

## Abstract

Large Conductance Voltage- and Calcium-activated K^+^ (BK) channels are transmembrane pore-forming proteins that regulate cell excitability and are also expressed in non-excitable cells. They play a role in regulating vascular tone, neuronal excitability, neurotransmitter release, and muscle contraction. Dysfunction of the BK channel can lead to arterial hypertension, hearing disorders, epilepsy, and ataxia. Here, we provide an overview of BK channel functioning and the implications of its abnormal functioning in various diseases. Understanding the function of BK channels is crucial for comprehending the mechanisms involved in regulating vital physiological processes, both in normal and pathological conditions, controlled by BK. This understanding may lead to the development of therapeutic interventions to address BK channelopathies.

## 1 Introduction

The large-conductance calcium- and voltage-activated potassium (BK; also known as MaxiK, K_Ca_1.1, Slo1) channel, encoded by the *KCNMA1* gene, is a transmembrane protein with a large conductance and exceptional selectivity for potassium. This channel is widely distributed in excitable cells of the central nervous system (CNS) and muscle and is also present in non-excitable tissues such as salivary, bone and kidney. Its activity have an influence on the shape, frequency, and propagation of action potentials (APs) and plays a critical role in synaptic function and neurotransmitter release (Contet et al., 2016; Latorre et al., 2017; Ancatén-González et al., 2023). The BK channel’s ability to selectivity mediate potassium extrusion leads to neuron repolarization and hyperpolarization, protecting against hyperexcitability and potential cell damage (Galarraga et al., 2007; Griguoli et al., 2016).

From worms to mammals, BK channels regulate excitability, Ca^2+^ signaling and the efflux of K^+^. In the circulatory system, they are vital for maintaining the membrane potential in vascular smooth muscle (VSM) cells. Abnormal functioning in VSM triggers alterations in vascular tone and can lead to hypertension (Wu and Marx, 2010; Contet et al., 2016). Smooth muscle BK channels significantly affect erectile function in the corpus cavernosum of the penis, playing a protagonist role in male physiology (Werner et al., 2005; Kun et al., 2009; Diniz et al., 2020). BK channels are also important in regulating airway muscle contractility by modulating Ca^2+^ influx in tracheal smooth muscle cells (SMC) in the respiratory system. In the gastrointestinal system, BK channels are essential for the motility of SMC, membrane depolarization and slow waves of Ca^2+^ from the interstitial cells of Cajal. (Lees-Green et al., 2011; Beyder and Farrugia, 2012; Sanders et al., 2012).

Furthermore, BK channels are critical for regulating extracellular potassium in the renal system. There is increasing evidence that BK channel activators may reduce renal fibrosis and improve kidney function (Yan et al., 2022). Additionally, alterations in the expression of auxiliary subunits of the BK channel have been linked to potassium retention and hyperkalemia (Grimm et al., 2009).

BK channels co-exist in microdomains with voltage-gated Ca^2+^ (Ca_v_) channels, Ca^2+^-permeable receptors and store-operated Ca^2+^ release channels, which connect BK channel activation in highly localized Ca^2+^ dynamics. An increase in intracellular Ca^2+^ activates BK channels, allowing K^+^ outflow and resulting in membrane hyperpolarization (Marty, 1981; Tricarico et al., 1997; Wu and Marx, 2010; Gutzmann et al., 2019; Yin et al., 2019). This is a crucial aspect for various physiological processes. The partnership between BK and Ca^2+^ channels has critical implications of physiological and pathophysiological mechanisms that affect BK channel gating with Ca^2+^.

Besides being present in the plasma membrane, BK channels can be found and play critical physiological roles in mitochondrial (mitoBK) and nuclear (nBK) membranes (for reviews, see Li and Gao, 2016; González-Sanabria et al., 2021). MitoBK channels, located in the inner mitochondrial membrane (IMM), are present in different cells and tissues, including mammalian ventricular myocytes, astrocytes, and endothelial cells. MitoBK shares most of its biophysical and pharmacological properties with plasma membrane BK (pmBK) channels. Notably, in adult cardiomyocytes, mitoBK is targeted to the IMM and is absent in the plasma membrane. Although the mitoBK is coded by the *KCNMA1* gene as the pmBK, mitoBK is a splice variant containing a C-terminal DEC splice insert that determines mitochondrial targeting (Singh et al., 2013). MitoBK plays a role in protecting the heart from ischemic injury and regulates the mitochondrial Ca^2+^ retention capacity (Singh et al., 2013; Li and Gao, 2016). The addition of Ca^2+^ or the BK channel agonist NS1619 to isolated brain mitochondria led to a mitochondria membrane potential depolarization and an increase in membrane respiration (Skalska et al., 2009)

nBK also shares most of the biophysical and pharmacological properties with pmBK (Li and Gao, 2016; González-Sanabria et al., 2021). nBK is present in the nuclear envelope of pancreatic cells, brain endothelial cells and macrophages. Located in the outer nuclear membrane, nBK controls the nuclear Ca^2+^ influx since nBK block with paxilline increases the nuclear Ca^2+^ (Li et al., 2014). Importantly, nBK are involved in nuclear Ca^2+^ signaling and the regulation of transcription factor activity (Li et al., 2014).

This review examines the physiology and clinical implications of dysregulated pmBK channels. BK channels have implications that span various medical fields and pathophysiological conditions beyond their basic functional properties. Understanding the mechanisms of BK channels in normal and abnormal conditions can illuminate their potential as therapeutic targets for numerous diseases. This article aims to provide readers with a perspective on the role of BK channels in cellular communication and human health.

## 2 Structure and activation of BK channels by Ca^2+^ and voltage

**FIGURE 1:**
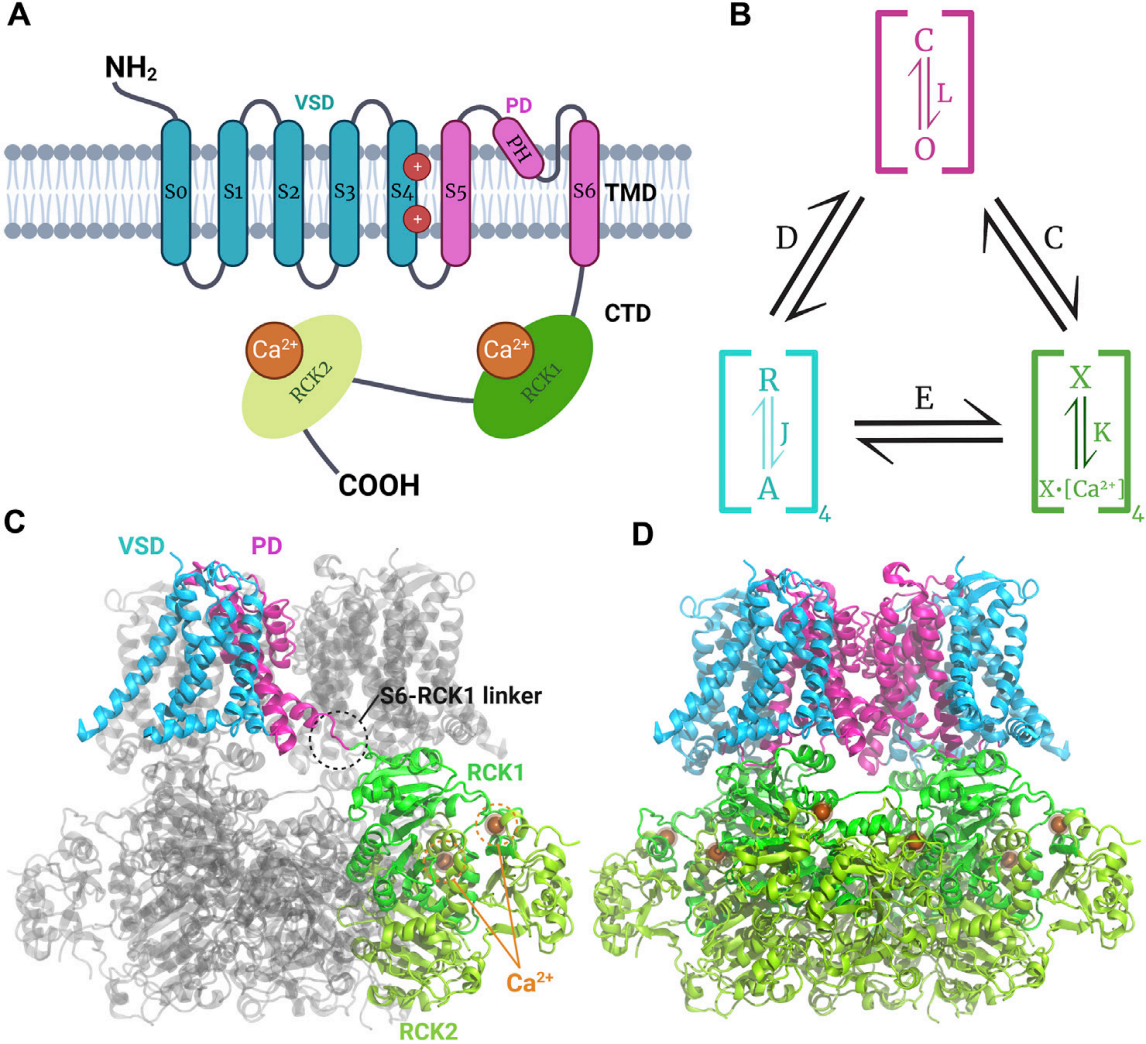
Model of structure and function of the BK channel. **(A)** Topology of monomeric BK channel α subunit showing the extracellular N-terminal region, the transmembrane domain (TMD) composed by the voltage sensor domain (VSD) where gating charges (R210 and R213 in the segment S4) reside (Carrasquel-Ursulaez et al., 2022) the pore domain (PD), and the intracellular C-terminal domain (CTD) where the RCK1 and RCK2 Ca^2+^ binding sites reside. **(B)** Schematic H-A allosteric gating model for BK channel activation by voltage and calcium (Horrigan and Aldrich, 2002). The PD can exist in close **(C)** or open (O) configurations defined by the equilibrium constant L. Each of the four VSD could be at rest (R) or activated **(A)** governed by the equilibrium constant J. The Ca2+ sensors may have calcium bound (X-Ca^2+^) or unbound (X) dominated by the equilibrium constant K. Each module is allosterically coupled to pore opening, governed by the indicated equilibrium constants The C for CTD-PD coupling, D for VSD-PD coupling, and E for VSD-CTD coupling. **(C, D)** Human BK channel cryo-EM Ca^2+^-bound structure (PDB:6V38) showing the non-domain swapped disposition of VSD and PD, whereas CTDs have a domain-swapped configuration forming the gating ring; the S6-RCK1 linker connects TMD and CTD.

BK channels belong to the voltage-dependent K^+^ channel (K_v_) superfamily (Latorre et al., 2010). The *KCNMA1* gene encodes the homotetrameric BK channel, which consists of an α subunit with seven transmembrane segments (S0-S6), an extracellular N-terminal region, and a C-terminal domain (CTD) facing intracellularly (Figure 1A). The CTD contains two regulatory domains of the K^+^ conductance (RCK1 and RCK2), which house the Ca^2+^ binding sites (Latorre et al., 2017). Segments S0-S4 comprise the voltage sensor domain (VSD), while segments S5-S6 form the pore domain (PD). BK channel has a substantially larger unitary conductance than other members of the voltage-activated K^+^ channel family, approximately 250 pS in symmetrical 100 mM K^+^. It also displays high selectivity for potassium (Latorre and Miller, 1983). The channel’s activity is regulated by the intracellular Ca^2+^ concentration and the membrane potential, which can activate the channel in a concerted or independent manner (Marty, 1981; Pallotta et al., 1981; Latorre et al., 1982; Eisenman et al., 1986). The channel’s activity can be modulated by auxiliary β or γ subunits, expressed in various cells and tissues, resulting in different channel phenotypes and pharmacological properties. For example, β4-containing BK channels are resistant to the scorpion toxin iberiotoxin (IbTX), and 17β-estradiol activates only BK channels formed by αβ complexes (β1, β2, β4), but not BK channels formed by the α subunit alone (Torres et al., 2014).

The BK channel’s characteristics enable it to play a pivotal role in modulating multiple cellular processes and excitability (Contet et al., 2016). Unlike K_v_ channels, the BK channel tetramer has a non-swapped configuration between the VSD and the PD, with the VSD contacting the PD of the same subunit. Additionally, the CTD of each subunit is swapped intracellularly, forming the gating ring (Figures 1C, D) (Tao et al., 2017). A comparison of the apo- and Ca^2+^-bound structures revealed that channel opening is induced by the expansion of the gating ring formed by the four CTDs upon Ca^2+^ binding (Hite et al., 2017; Tao et al., 2017). The structures of the BK channels in complex with the β4 and γ1 subunit reveal an α: β4 and α: γ1 = 1:1 stoichiometry (Tao and MacKinnon, 2019; Yamanouchi et al., 2023). αβ4 BK channels have a structure similar to that of channels formed by the α subunit alone, suggesting that the β4 modulates the stability of pre-existent structures (Tao and MacKinnon, 2019). The structure of the αβ4 BK channel shows that the external loop joining together the two transmembrane segments of the β4 subunit forms a crown on top of the external aspect of the channel, which constrains the access of the toxin to its binding site. The recently solved structure of the γ1 α BK channel indicates that this subunit, through its transmembrane segment and the arginine cluster present in its C-terminal, stabilizes the active conformation of voltage and Ca^2+^ sensors, respectively (Yamanouchi et al., 2023).

The weak voltage dependence of the BK channel compared to other K_v_ channels can be explained by the lower number of gating charges involved in channel activation. In the BK channel, the gating charges are two arginine (R210 and R213) residues located in segment S4. The BK gating charges do not move across the entire electric field, as occurs with the gating charges implicated in K_v_ voltage activation (Carrasquel-Ursulaez et al., 2022).

The communication between the VSD, the Ca^2+^ binding sites, and the pore gate is allosteric. The BK channel can open when all voltage sensors are at rest and without internal Ca^2+^, although with a very low probability of opening (P_O_). The free energy required to open the pore decreases as voltage sensors activate and Ca^2+^ binds to the high-affinity sites in the RCKs. Figure 1B presents a schematic model of allosteric gating kinetics that considers the channel’s tetrameric structure, where the different channel modules are coupled by allosteric factors D, C, and E (Horrigan and Aldrich, 2002). It is important to note that, in this model, the channel can be activated independently by internal Ca^2+^ and depolarizing voltages. This model has successfully explained BK channel gating, the effects of different auxiliary subunits, and BK channelopathies (Latorre et al., 2017).

## 3 Functional diversity of the BK channel in the nervous system

**FIGURE 2:**
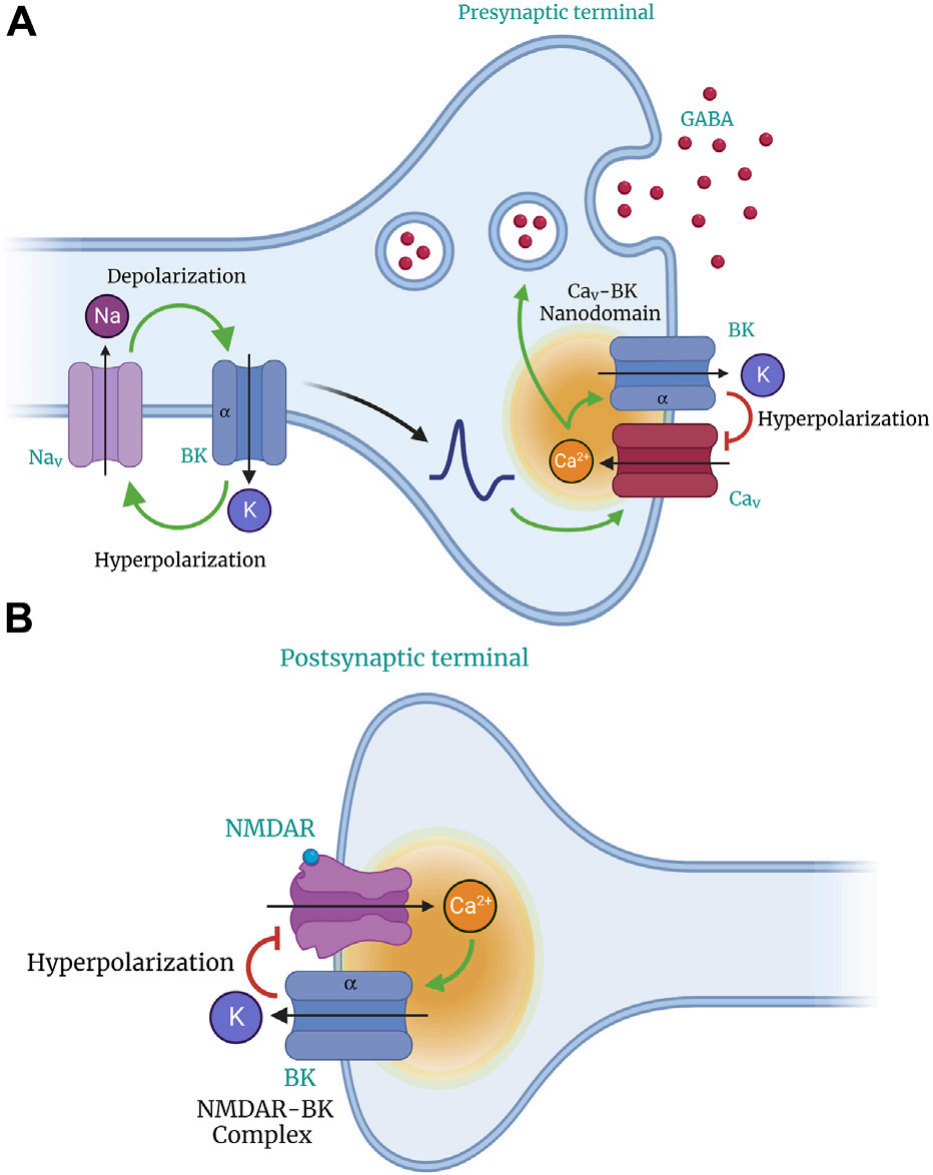
Role of BK channels in neuronal excitability and neurotransmitter release. BK channels act as feedback regulators to control neuronal excitability. Their activation caused by depolarization results in a massive efflux of K^+^ ions, hyperpolarizing the cell membrane. Through their voltage sensitivity and ability to detect changes in Ca^2+^ concentration, activation of BK channels modulates neuronal excitability and limits transmitter release (GABA). BK channels co-localize and form nanodomains with voltage-gated Ca^2+^ (CaV) channels in presynaptic compartments **(A)** and N-methyl-D-aspartate (NMDA) receptors in postsynaptic terminals **(B)**. The co-localization of BK and CaV channels provides BK channels with an effective local Ca^2+^ concentration for their activation and function.

The BK channel is a coincidence detector that synergistically responds to Ca^2+^ and voltage (Latorre et al., 1989). It its located in membrane microdomains near voltage-gated Ca^2+^ (Ca_v_) channels (Horrigan and Aldrich, 2002) (Figure 2), which allows it to regulate the activity of excitable cells through negative feedback control of Ca^2+^ influx via voltage-activated Ca_v_ channels. BK channels play a crucial role in the rapid repolarization of excitable cell membranes. Despite their weaker voltage dependence than other K_v_ channels (Latorre et al., 1989), BK channels activate and deactivate rapidly under physiological conditions and do not inactivate under sustained depolarization (Faber and Sah, 2003).

Although a single gene (*KCNMA1*) encodes the pore-forming BK channel α subunit, phenotypic diversity is achieved through alternative splicing, interaction with auxiliary β- and γ subunits, and metabolic regulation (Sun et al., 1999; Orio et al., 2002; Fodor and Aldrich, 2009; Sitdikova et al., 2014; Torres et al., 2014; Latorre et al., 2017; Carrasquel-Ursulaez et al., 2018; Sánchez-Aguilera et al., 2020; Carrasquel-Ursulaez et al., 2022). There are four auxiliary β subunits (β1–β4) of the BK channel, encoded by four genes (*KCNMB1-KCNMB4*). They share a common structure of two transmembrane segments connected by a large extracellular loop and cytoplasmic COOH and NH_2_ termini. All β subunits modify BK channels’ gating and pharmacological properties to various degrees. The compartmentalized distribution of β subunits in neurons may have important functional implications for understanding how BK channels differentially regulate the excitability of somatic and synaptic terminal compartments (Knaus et al., 1994; Brenner et al., 2000; Uebele et al., 2000; Contreras et al., 2012; Kyle and Braun, 2014).

β4 is the most abundant auxiliary BK subunit expressed in the nervous system (Wallner et al., 1999; Uebele et al., 2000). Its distribution largely overlaps the α subunit, with prominent expression in the thalamus, brainstem, posterior pituitary terminals, cortical pyramidal neurons, hippocampal CA3 pyramidal neurons, hippocampal dentate granule cells, olfactory bulb, cerebellar Purkinje cells (PCs), spinal cord, and trigeminal motor nuclei (Wallner et al., 1999; Uebele et al., 2000; Piwonska et al., 2008). The BK(α+β4) channel displays slow gating kinetics, compared with α alone, and is insensitive to classical BK blockers, such as iberiotoxin (IbTX) and charybdotoxin (ChTX) (Wang et al., 2006). Previously, αβ4BK channels were classified as a subtype of BK channels known as “type II”. The presence of β4 significantly slows BK channel activation kinetics (Petrik and Brenner, 2007), similar to the effect caused by the β1 and β2 subunits (Orio et al., 2002; Orio and Latorre, 2005). It is important to note that the β4 KO has the effect of converting the BK channel from type II (slow gating) to type I (fast gating), which results in a decrease in the duration of action potentials (Reinhart et al., 1989; Weiger et al., 2000).

αβ2BK channels exhibit fast inactivation in adrenal chromaffin cells. The inactivation process is conferred by the N-terminal residues of the β2 subunit (Solaro and Lingle, 1992; Wallner et al., 1999; Xia et al., 1999). This subunit is expressed in the brain and has been identified in hippocampal, neocortical, and lateral amygdala pyramidal neurons, as well as in (PCs), and dorsal root ganglion (DRG) neurons (Knaus et al., 1994; Brenner et al., 2000; Horrigan and Aldrich, 2002; Contreras et al., 2012; Whitt et al., 2016; Latorre et al., 2017; Ancatén-González et al., 2023).

The β1 subunit has been detected in the hypothalamus, paraventricular neurons and cerebellar PCs, and is highly abundant in cerebral artery myocytes (Tseng-Crank et al., 1996; Jiang et al., 1999; Salzmann et al., 2010). The association of BK channels with the β1 subunit makes them a target for alcohol-induced potentiation of their activity (Feinberg-Zadek et al., 2008). The β3 subunit exhibits weak expression in the CNS (Brenner et al., 2000; Contreras et al., 2012).

The BK channel auxiliary subunits include a γ (γ1–γ4) family. A comparative analysis of the distribution of γ subunits has shown that γ3 is selectively enriched in the brain, while γ1 and γ4 are expressed at lower levels. In contrast, γ2 is essentially absent in this organ (Li and Yan, 2016). αγ1BK channels in cerebral arterial myocytes increase the voltage and Ca^2+^ sensitivity of BK channels, thereby reducing myogenic tone and promoting vasodilation (Contet et al., 2016). The role of γ3 in modulating neuronal physiology remains to be determined.

### 3.1 The BK channel expression in the central nervous system

BK channels are expressed extensively throughout the CNS, including the cortex, basal ganglia, hippocampus, thalamus, and cerebellum, among other regions (Contet et al., 2016; Latorre et al., 2017; Ancatén-González et al., 2023). The BK channel α subunit is upregulated in the CNS during late embryonic and early postnatal development (Hafidi et al., 2005; MacDonald et al., 2006). BK currents exhibit a sudden increase during the first 2 weeks after birth in neocortical pyramidal neurons, substantia nigra dopamine neurons, and cochlear inner hair cells (Kang et al., 1996; Langer et al., 2003; Marcotti et al., 2003; Ramírez-Latorre, 2012). BK channel expression is regulated during development in hippocampal neurons and contributes to the repolarization of single-action potential during the first postnatal week (Hunsberger and Mynlieff, 2020). In contrast, BK currents are transiently reduced during adolescence in medial prefrontal cortex pyramidal neurons (Benhassine and Berger, 2005; Ksiazek et al., 2013). This developmental pattern suggests that BK channels shape the properties of mature neuronal firing and contribute to experience-dependent plasticity (Contet et al., 2016).

In the postnatal central nervous system, BK channels are located in the plasma membrane of neurons and influence the shape, frequency, and propagation of APs, thus controlling synaptic function and neurotransmitter release from presynaptic terminals (Vilchis et al., 1999; Benhassine and Berger, 2005; Goldberg and Wilson, 2005; Galarraga et al., 2007; Gittis et al., 2010; Alle et al., 2011; Kohashi and Carlson, 2014; Kimm et al., 2015; Bock and Stuart, 2016; Griguoli et al., 2016). BK channels are expressed in various neuronal compartments, including axons, dendrites, synaptic terminals, fiber tracts, and somatodendritic compartments (Knaus et al., 1996; Hu et al., 2001; Raffaelli et al., 2004; Sailer et al., 2006; Benhassine and Berger, 2009; Hirono et al., 2015; Ancatén-González et al., 2023), where they perform distinct functions. (Benhassine and Berger, 2005; Benhassine and Berger, 2009; Gómez et al., 2021; Tazerart et al., 2022). Additionally, BK channels are present in mitochondria, lysosomes, and the nuclear envelope of neurons, where they affect gene transcription and neuronal morphology (Gu et al., 2014; Li and Gao, 2016; González-Sanabria et al., 2021). In addition, the channel is expressed in non-neuronal cell populations, such as astrocytes and vascular SMC, which regulate cerebral blood flow. BK channels play a pleiotropic role in regulating the activity of neural circuits in the brain and spinal cord by providing a negative feedback mechanism for local increases in intracellular Ca^2+^ concentration (Contet et al., 2016) (see Figure 2).

### 3.2 BK channels in neuronal excitability

The relationship between neuronal activity levels and the influence of BK channels on AP firing rate is associated with the co-expression of other voltage-activated currents. BK channels co-localize and form nanodomains with L,P/Q,N, R, or T-type Ca_v_ channels and N-methyl-D-aspartate receptors (NMDAR) (see Figure 2) (Berkefeld et al., 2010; Rehak et al., 2013; Bellono et al., 2017; Shah et al., 2022). The co-localization of BK and Ca_v_ channels provides BK channels with an effective local Ca^2+^ concentration for their activation and function (Naraghi and Neher, 1997; Berkefeld and Fakler, 2008; Rehak et al., 2013; Shah et al., 2022). During the propagation of an action potential, the activation of Ca_v_ channels by membrane depolarization modifies the voltage dependence, current amplitude, and kinetics of BK channel activation. This results in the functional diversity of BK channels (Berkefeld et al., 2006; Vivas et al., 2017). The activation of BK channels in the presynaptic region reduces the duration of AP, promotes fast hyperpolarization potentials, shortens the time of Ca^2+^ influx, and limits neurotransmitter release (Figure 2A) (Edgerton and Reinhart, 2003; Müller et al., 2007; Gutzmann et al., 2019). BK channels act as feedback regulators in the nervous system, serving as an “emergency break” that prevents transmitter-induced hyperexcitability and associated cell toxicity (Bentzen et al., 2014). Their large conductance leads to neuronal repolarization, hyperpolarization, and afterhyperpolarization (AHP), which decreases cellular excitability (Shao et al., 1999; Womack and Khodakhah, 2002; Salkoff et al., 2006; Galarraga et al., 2007; Griguoli et al., 2016; Hunsberger and Mynlieff, 2020; Niday and Bean, 2021).

In contrast to BK channels that are closely associated with Ca_v_ channels, BK channels that do not form nanodomains with Ca_v_ channels may play a role in sensing global Ca^2+^ concentration and provide an “emergency brake” under excitotoxic conditions where [Ca^2+^]_i_ rises above the physiological range (Hu et al., 2001; Rundén-Pran et al., 2002).

**FIGURE 3:**
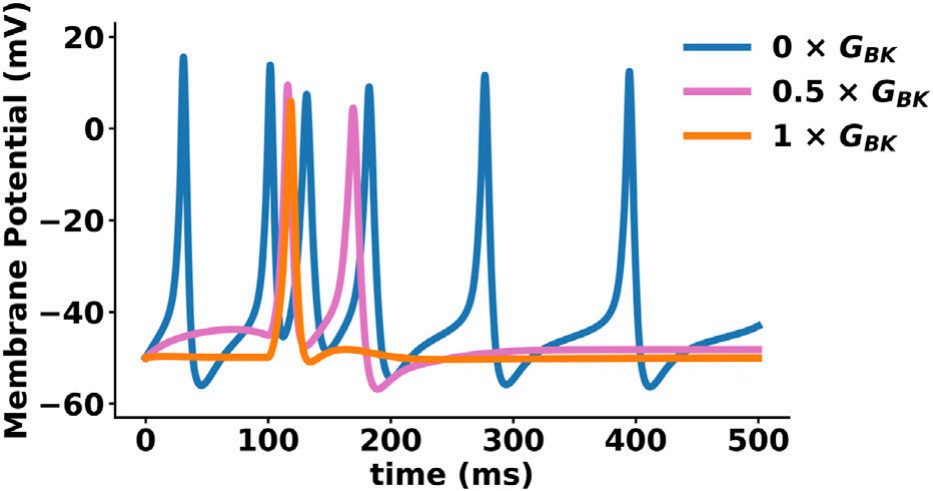
Effect of BK channel function on action potential (AP) shape, size, and firing rate. AP simulation using NEURON (Mahapatra et al., 2018). This model considers multiple ionic conductances, including calcium channels (L-type and T-type), voltage-gated potassium channels (Kv1, KCNQ), ATP-activated potassium channels, voltage- and calcium-activated potassium channels (BK, SK, IK), and a leak current. The plot shows three APs with different percentages of BK conductance (0%, 50%, 100%), exemplifying BK channel role in APs firing frequency. A reduction in BK channel conductance (GBK) increases smooth muscle cell excitability and may contribute to pathological conditions such as hypertension, erectile dysfunction (ED), and overactive bladder (OAB).

BK channels can have complex effects on firing frequency and the shape of the AP waveform, as shown in Figure 3 (Horrigan and Aldrich, 2002; Gu et al., 2007; Womack et al., 2009; Hull et al., 2013; Mahapatra et al., 2018). Inhibition of BK channels, either through pharmacological or genetic means, has been show to regulate the timing and duration of K^+^ influx, resulting in changes to evoked spontaneous AP firing in CNS (Behrens et al., 2000; Jin et al., 2000; Du et al., 2005; Nelson et al., 2005; Meredith et al., 2006; Pitts et al., 2006; Gu et al., 2007; Gan et al., 2008; Matthews et al., 2008). It is important to note that the effects of BK channels on firing activity can be both excitatory and inhibitory. This finding supports the idea that BK channels should not be considered strictly excitatory or inhibitory. Instead, they decrease the firing of overactive neurons and increase the firing of quiescent neurons (Contet et al., 2016). In neurons, currents from other ion channels and cell-specific factors determine whether BK channels contribute to the repolarization or AHP phases of the AP. As a result, BK currents can either slow or accelerate spontaneous firing (Gu et al., 2007). Thus, the functional role of the BK channel is dependent on the presence of other conductance within the cell, as well as their specific location and composition (Ancatén-González et al., 2023).

In certain types of neurons, such as Purkinje Cells (PC), vestibular, and cerebral Golgi cells, the inhibition of BK channels leads to an acceleration of AP firing, accompanied by a reduction in AHP (Womack and Khodakhah, 2002; Galarraga et al., 2007; Gittis et al., 2010; Hull et al., 2013; Niday and Bean, 2021). Conversely, in hippocampal CA1 pyramidal, GABAergic, and intracardiac autonomic neurons, the inhibition of BK channels slows the firing rate (Gu et al., 2007; González-Corrochano et al., 2013; Pérez et al., 2013). Inhibition of BK channels in the substantia nigra pars compacta results in a pronounced broadening of the AP, a more negative AHP, and an increase in firing rate (Kimm et al., 2015). In the vestibular nucleus (with higher basal firing) and PCs, pharmacological inhibition of BK channels with IbTX increases firing rates and decreases AHP (Smith et al., 2002; Womack et al., 2009). These observations suggest that BK channels preferentially contribute to either the repolarization phase of the AP or the AHP phase (Contet et al., 2016).

Increasing evidence suggests that BK channels are present in dendritic compartments, which modulate neuronal excitability (Benhassine and Berger, 2005; Grewe et al., 2010; Tazerart et al., 2022; Ancatén-González et al., 2023). In the somatosensory cortex, BK channels are present in apical dendrites and suppress Ca^2+^ channels to regulate Ca^2+^ spikes and dendritic excitability (Grewe et al., 2010). In layer five pyramidal neurons, BK suppresses the backpropagation of AP during high-frequency firing and increases the threshold for dendritic spiking (Golding et al., 2002; Benhassine and Berger, 2005). This backpropagation can amplify dendritic APs, facilitating the induction of long-term potentiation and thus contributing to synaptic plasticity (Golding et al., 2002; Holthoff et al., 2004). Conversely, BK channels also restrict dendritic Ca^2+^ spikes both temporally and spatially in CA1 pyramidal cells, inhibiting synaptic potentiation (Filosa et al., 2006). Inhibition of BK channels has been demonstrated to enhance the propagation of dendritic Ca^2+^ spikes in cerebellar PCs, facilitating a form of short-term synaptic plasticity at distant spines (Golding et al., 2002). BK-mediated effects on dendritic spike generation may depend on the size of the spine at which they are expressed. BK channels are only activated in small spines of basal dendrites of V-layer pyramidal neurons in the visual cortex. This activation suppresses excitatory postsynaptic potentials (Tazerart et al., 2022).

The suprachiasmatic nucleus (SCN) of the hypothalamus expresses BK channel, which acts as a master pacemaker driving the circadian control of physiology and behavior in mammals (Montgomery and Meredith, 2012). The BK channel regulates the magnitude and duration of the AHP, controlling the circadian rhythm (Armstrong and Roberts, 2001; Whitt et al., 2016). During the day BK currents are low, but they increase at night. Additionally, the average spontaneous firing rate of SCN neurons decreases during the night. The BK channel plays a crucial role in determining the SCN firing rate, which is essential for normal circadian timing. During the day, the β2 subunit confers N-type inactivation of the BK channel, resulting in large BK currents. This inactivation process decreases during the night, leading to the predominance of BK currents. The absence of β2 subunit in KO mice eliminates this variation, indicating that the BK inactivation process acts as a switch to set the circadian variations in SCN excitability (Whitt et al., 2016).

### 3.3 The role of BK channels in neurotransmitter release

BK channels regulate excitatory and inhibitory transmitter release, contributing to the excitatory-inhibitory balance necessary for normal brain function (Ancatén-González et al., 2023). Ca^2+^ entry at the presynaptic terminal through Ca_v_ channels activated in response to AP serves as a signal to initiate exocytosis of neurotransmitter-containing vesicles and activate local BK channels (Regehr and Kaeser, 2014) (see Figure 2A). Generally, presynaptic BK channel activation acts as a negative regulator of neurotransmitter release by hyperpolarizing the plasma membrane (Wang, 2008). Pharmacological blockade of BK channels has demonstrated their influence on neurotransmitter release of BK channels at neurosecretory terminals, neuromuscular junctions and central synapses, resulting increasing vesicle secretion (Obaid et al., 1989; Robitaille and Charlton, 1992; Robitaille et al., 1993; Dopico et al., 1999). BK channels contribute to GABA release from retinal A17 amacrine cells (Figure 2A), layer II/III interneuron pyramidal neurons, and the central amygdala (Grimes et al., 2009). BK channels regulate inhibitory glycine release in the spinal cord (De San Martín et al., 2010) and acetylcholine (ACh) release from efferent terminals of the medial olivocochlear system to inner hair cells (Zhang Y. et al., 2014). CNS disorders such as epilepsy, ataxia, intellectual disability, and chronic pain have been associated with both BK channel loss and gain of function (Contet et al., 2016; Cui, 2021). BK channels can potentially be exploited to correct neurological dysfunction in various pathological conditions (Contet et al., 2016).

### 3.4 BK channels in non-neuronal cells of the CNS

BK channels play a significant role in non-neuronal cell types of the CNS, including astrocytes and microglial cells. In astrocytes, BK causes vasodilation and is associated with astrocyte terminal processes surrounding parenchymal blood vessels. BK channel activity is also critical role in the rapid vasodilation of intracerebral arterioles produced by adjacent neuronal activity (Paisansathan et al., 2010). Similarly, BK channels located in the glial limiting membrane, which is the barrier of astrocytic endfeet surrounding the brain and spinal cord, participate in the vasodilation of pial arterioles. This vasodilation plays a fundamental role in regulating cerebral blood flow. Activation of BK channels hyperpolarizes arteriolar SMCs, causing local vasodilation (Bordey and Spencer, 2003; Girouard et al., 2010; Paisansathan et al., 2010; Hayashi et al., 2011).

BK channels are found in microglial cells in various regions of the brain, including the hippocampus, striatum, neocortex, and entorhinal cortex, as well as in the spinal cord following nerve injury (Buttigieg et al., 2011; Regehr and Kaeser, 2014; Schilling and Eder, 2015; Sun et al., 2023). Microglia are immune cells that reside in the brain, and the activity of BK channels regulates their phagocytosis activation and migration (Larson et al., 2016). BK channels are also present in oligodendrocyte precursors (Wulf et al., 2008; Poulsen et al., 2009), where they regulate Ca^2+^ influx. BK channel expression gradually decreases during the maturation of precursors into oligodendrocytes, suggesting that BK currents may play a role in the differentiation of these cells (Poulsen et al., 2009). Furthermore, BK channels are present in cerebral blood vessels and meninges (Vetri et al., 2014; Krishnamoorthy-Natarajan and Koide, 2016). Like other blood vessels, BK channels are crucial in promoting vasodilation of cerebral arteries in response to Ca^2+^ sparks from the sarcoplasmic reticulum (Stojilkovic et al., 2005).

## 4 BK channel in the endocrine system

BK channels are found in various cells of the endocrine system, including the acinar cells of the submandibular and parotid glands (Venglovecz et al., 2011). The apical membrane of lacrimal acinar cells has a high density of BK channels, which aids in electrolyte secretion from this gland (Lee et al., 2012). Further, BK channels are present in pancreatic cells on both the basolateral and luminal membranes of the pancreatic duct epithelial cells, which secrete a pancreatic juice rich in HCO_3_ (Kros and Crawford, 1990; Duncan and Shipston, 2016; Whitt et al., 2018). The opening of BK channels facilitates the appropriate driving force for HCO_3_ secretion from the luminal membrane (Kros and Crawford, 1990; Dulon et al., 1995). However, in certain anterior pituitary cell types, these channels cause bursting activity, having an excitatory effect (Rao et al., 2017). Conversely, in the posterior pituitary, BK channel activation reduces the excitability of neuroendocrine cells, leading to the inhibition of hormone secretion. The precise role of BK channels in controlling pituitary cell excitability is still unknown (Rüttiger et al., 2004).

BK channels are expressed in melanotrophs (Kehl and Wong, 1996) and lactotrophs (Vacher et al., 1998), GH3, and AtT-20 pituitary cells (Lang and Ritchie, 1990; Van Goor et al., 2001; Mahmoud and McCobb, 2004; Contreras et al., 2013). The BK channel current contributes to membrane repolarization during the downstroke of an AP, which is critical to the bursting behavior in a fraction of somatotrophs. Higher expression of BK channel in these cells is responsible for the different activity patterns of somatotrophs, lactotrophs, and gonadotrophs (Kanyicska et al., 1997; Rao et al., 2017).

Glucocorticoids regulate alternative splicing of the BK α subunit in the adrenal and pituitary glands. In AtT-20 cells, glucocorticoids rapidly activate BK channels via serine/threonine protein phosphatase. The sensitivity of the BK channel to glucocorticoids is determined by the BK e21 splice variant being predominantly expressed in endocrine tissues (Sitdikova et al., 2010). These hormones alter the channel’s ability to respond to Ca^2+^, oxidation, and phosphorylation (Lang and Ritchie, 1990; King et al., 2006; Latorre et al., 2017). BK e21 increases the channel’s apparent Ca^2+^ sensitivity. Additionally, gonadal testosterone plays a role in regulating alternative splicing of the Slo1 α subunit in pituitary cells (Wu et al., 2007). Stress affects the splicing decision and mRNA expression levels of the α-, β2-, and β4 subunits (Maruyama et al., 1983). Steroid hormones regulate the β2-and β4 subunits of BK channels (Tan et al., 1992). BK channels contribute to AP repolarization in GH3 cells, primarily terminating AP firing (Lang and Ritchie, 1990). These cells are commonly used to study the modulation of native BK channels by various compounds (Gray et al., 1990; Hede et al., 2005).

## 5 The BK channel in the auditory system

The anatomy of the mammalian cochlea and the organ of Corti and the mechanical properties of the basilar membrane contribute to the spatial separation of sound frequencies. This tissue organization allows for tonotopic or frequency-place mapping, where specific frequencies stimulate localized hair cells. BK channels, present in both inner and outer hair cells contribute significantly to the total potassium current. Therefore, it is a crucial target for function in the mammalian sensory epithelium and contributes to outward potassium conductance playing a critical role in mammalian hearing (Fettiplace, 2023).

BK channels have been identified in isolated guinea pig inner hair cells (Kros and Crawford, 1990). The presence of the BK channel in this cell type has been confirmed by non-selective inhibitors such as tetraethylammonium ion (TEA), and specific inhibitors such as IbTX and ChTX (Dulon et al., 1995; Hafidi et al., 2005). Electrophysiological recordings and RT-PCR have also identified the α subunit of the BK channel in the organ of Corti in rats (Brändle et al., 2001). The significance of the BK channel in progressive hearing loss has been demonstrated in KO mice. However, the KO of the β1 subunit does not result in hearing loss (Rüttiger et al., 2004). In frog hair cells and mouse inner hair cells, the β2 subunit and specific splice variants of the β3 subunit have been found to induce inactivation of BK channel currents, which may contribute to BK channel inactivation (Armstrong and Roberts, 2001). Additionally, auxiliary γ subunits may contribute to the distinctive characteristics of BK currents in rat inner hair cells. Specifically, γ2 has been shown to activate BK currents at negative membrane potentials independently of Ca^2+^ (Lang et al., 2019). Inner hair cells from γ2 KO mice display a rightward shift in the voltage dependence of BK channel activation by more than 200 mV, along with a disruption in BK localization (Armstrong and Roberts, 2001). The removal of γ2 caused a significant change in BK channel activation, exceeding the −90 mV leftward shift observed when co-expressing γ2 with the BK channel α subunit. This suggests that γ2 stabilizes a macromolecular complex required activating the BK conductance at negative membrane potentials and for proper channel localization (Lingle et al., 2019).

Electrophysiological recordings have confirmed the presence of BK channels in the outer hair cells of guinea pigs (Spreadbury et al., 2004). In addition, α and β1 subunits have been identified in outer hair cells of the guinea pig organ through *in situ* hybridization and immunocytochemistry (Skinner et al., 2003; Rüttiger et al., 2004). Moreover, degeneration of outer hair cells has been observed in the basal and midbasal cochlear turns of BKα KO mice (Rüttiger et al., 2004), which also causes insensitivity in the high-frequency range of the cochlea (Engel et al., 2006).

## 6 BK channel in the respiratory system

**FIGURE 4:**
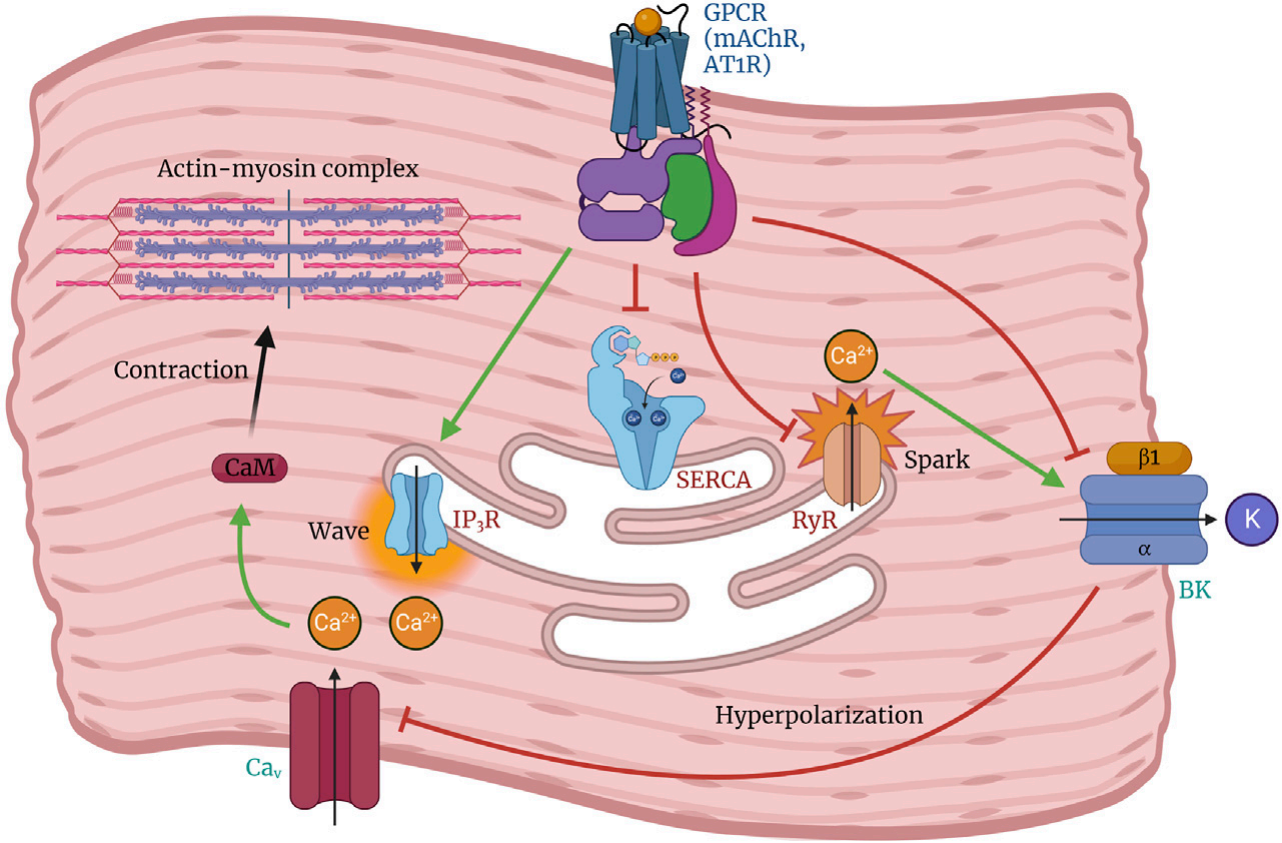
Role of the BK channel in regulating arterial tone and smooth muscle contraction. Depolarization of arterial smooth muscle cells by activation of G-protein coupled receptors (GPCRs) triggers the opening of Cav channels, leading to Ca^2+^ influx followed by sarcoplasmic reticulum Ca^2+^ release (Ca^2+^ wave) from the inositol triphosphate receptor (IP3R), which promotes cell contraction. Under conditions favoring vasodilation, Ca^2+^ release from the ryanodine receptor (RyR) is stimulated by luminal SR Ca^2+^ levels, resulting in a localized Ca^2+^ spark that activates the BK channel. This leads to membrane hyperpolarization and subsequent inhibition of Cav channel activity. The balanced interplay between BK, RyR, and Cav channels can promote relaxation or contraction depending on the regulation of their activity.

The activity of the BK channel in respiratory neurons affects the duration of AP firing patterns that regulate respiratory rate in mice (Bissonnette, 2002). The activation of BK channels plays a crucial role in modulating smooth muscle contractility (Bolton and Imaizumi, 1996; Hull et al., 2013; Gao et al., 2023) by facilitating spontaneous transient outward K^+^ currents (STOCs). These results in hyperpolarization of the cell membrane, leading to a decrease in Ca^2+^ influx and subsequent attenuation of smooth muscle activity. Tracheal smooth muscle contractions often occur after activation of inositol trisphosphate (IP_3_) receptors, which leads to Ca^2+^ release from intracellular Ca^2+^ stores (Figure 4). This phenomenon is commonly referred to as pharmacomechanical coupling. Semenov et al. (Semenov et al., 2006) demonstrated that the BK(α+β1) channel plays a fundamental role in controlling tracheal muscle contraction. The study indicates that decreased BK channel activity could result in heightened tracheal constriction upon exposure to cholinergic stimulation.

Benzbromarone is a uricosuric agent used to treat gout and hyperuricemia. It acts as an activator of BK channels, reducing the closed state duration and increasing their P_O_ (Gao et al., 2023). This drug also reduces the contraction of smooth muscle in the respiratory tract by inhibiting the entry of Ca^2+^ions through voltage-dependent Ca^2+^ channels, mitigating the constriction of the smooth muscle lining the respiratory airway (Gao et al., 2023).

## 7 BK channel in the gastrointestinal system

The activation of the BK channel is essential in the physiological processes related to gastrointestinal motility. Smooth muscle contraction in the gastrointestinal tract is initiated by the depolarization of SMCs caused by slow waves generated by ICC (Tricarico et al., 1997; Oshiro et al., 2005). This depolarization process triggers the activation of Ca_v_ channels, which allows the influx of Ca^2+^ and subsequently increases the concentration of the divalent cation within the cell. This increase in intracellular Ca^2+^ concentration leads to the contraction of SMCs (Shen et al., 2021).

The involvement of BK channel in the occurrence of spontaneous contractions in rat colonic smooth muscle was demonstrated through the use of carbachol, an agonist of muscarinic ACh receptors (mAChR) that can decrease BK channel activity (Xin et al., 2018). The administration of carbachol facilitates the contraction of colonic smooth muscle by activating mAChR. This activation enhances the activity of L-type Ca^2+^ channels, activates IP_3_, and inhibits the sarcoendoplasmic reticulum Ca^2+^ ATPase (SERCA) pump, leading to an increase in intracellular Ca^2+^ levels (Figure 4). The observed suppressive effect of carbachol on BK channels in specific physiological and pharmacological contexts suggests that activation of mAChR may interfere with BK channel function and enhance colonic motility (Xin et al., 2018).

## 8 BK channel in the vascular system

The relationship between the BK channel and regulatory factors is crucial in vascular physiology. BK channels are crucial in regulating vasomotor tone in the vascular system. They are essential for maintaining membrane potential and balancing intracellular Ca^2+^ and potassium concentrations. Any malfunction or aberration in BK channel activity can result in vascular tone alterations and pathological conditions, including hypertension (Ghatta et al., 2006; Carvalho-de-Souza et al., 2013). For instance, angiotensin II downregulates the BK channel in VSMCs through the protein kinase C (PKC) signalling pathway and in conjunction with the angiotensin II type 1 receptor (AT1R) receptor (Yin et al., 2019). Angiotensin II increases intracellular Ca^2+^ levels while inhibiting the production and activity of the BK channel (Figure 4) (Yin et al., 2019). This inhibition results in the phosphorylation of the AT1R-BKα heterodimer, leading to channel desensitization or internalization (Yin et al., 2019).

The significance of the BK channel in regulating VSM relaxation and maintaining vascular tone homeostasis is evident from studies utilizing BK channel inhibitors (Yin et al., 2019). The activation of the BK channel in arterial smooth muscle partially regulates the myogenic tone. TEA and ChTX, which block Ca^2+^-activated K^+^ channels, depolarize and constrict cerebral arteries. This effect is significantly reduced when intracellular Ca^2+^ is low or by blocking Ca_v_ channels. The BK channel functions as a negative feedback mechanism that regulates membrane depolarization and vasoconstriction. In VSMCs, the BK channel is co-expressed with the β1 subunit. Selective removal of the gene encoding β1 can alter the voltage and Ca^2+^ sensitivity of the BK channel, which may increase vascular tone or even hypertension (Brenner et al., 2000; Wu and Marx, 2010).

Regarding BK subunit diversity in the circulatory system, the α subunit is present in various cellular populations, such as endothelial cells and VSMCs. Additionally, the expression of β1 subunits is prominent in the heart and VSM but appears absent in the endothelium (Brenner et al., 2000; Eichhorn and Dobrev, 2007; Hu and Zhang, 2012).

## 9 BK channel in the renal system

BK channels are expressed in various cells and segments of the nephron and contribute to regulating renal function. They have been observed in podocytes (Morton et al., 2004) and glomerular mesangial cells and may play a role in controlling mesangial contractility and glomerular filtration rates (Stockand and Sansom, 1994). BK expression has been identified in almost all segments of the nephron (Meera et al., 2000), including the proximal and distal convoluted tubules (DCT), as well as in mammalian kidney cell lines such as Madin–Darby type-II canine kidney cells (Holtzclaw et al., 2010). Recent studies have also identified BK channels as mediators of flow-induced potassium secretion (FIKS) in the distal tubule and cortical collecting duct (CCD) (Kunau et al., 1974).

STREX transcripts have been identified in mammalian kidneys, but their specific role in potassium secretion in the potassium-secreting convoluted tubule or CCD segments is not fully understood (Lai and McCobb, 2002). Transcript analysis of the whole mouse kidney has found the auxiliary subunits β1, β2, and β4 (Grimm et al., 2007). Studies of specific cells and segments of the nephron have demonstrated the presence of β4 subunits in cultured human podocytes and rabbit CCDs (Pluznick et al., 2005). All β subunits may be expressed in different kidney areas. Localizing β subunits within specific cells provides valuable insights into the function of BK in these cells (Tricarico et al., 1997). The BK channel in the renal connecting tubule expresses the β2 subunit and deleting this subunit in mice has resulted in high renal potassium retention and hyperkalemia (Yan et al., 2022). Additionally, β4 KO have resulted in low potassium excretion, mild hypertension, and mild fluid retention in mice treated with a high-potassium diet (Grimm et al., 2009).

The BK channel is activated indirectly by shear stress in the kidney through coupling to TRPV4 channel activation and their role in Ca^2+^ influx in endothelial cells (Reiter et al., 2006). Recent observations have shown that some BK channel activators, such as NS1619 and BMS191011, attenuate renal fibrosis and promote proper kidney function in animal models (Yan et al., 2022).

## 10 BK channel in the reproductive system

The BK channels present in the smooth muscle of the corpus cavernosum of the penis have a significant impact on erectile performance. These channels are activated by an increase in intracellular Ca^2+^ concentration, which leads to hyperpolarization of smooth muscle cells. This physiological mechanism ultimately results in relaxation and vasodilation of the penile blood vessels. Hyperpolarization decreases cellular excitability, promoting increased blood flow to the corpora cavernosa and facilitating the erection process. The absence of BK channels in experimental models, such as Slo1 KO mice, has been linked to erectile dysfunction, highlighting the biophysical significance of these channels in regulating male sexual function (Werner et al., 2005). In the tests, BK regulates smooth muscle tone and blood flow. This regulation is essential for spermatogenesis and sperm maturation (Hristov et al., 2016). Moreover, BK channels induce membrane hyperpolarization in testis SMCs, thereby regulating sperm movement. The interplay between BK channels, intracellular Ca^2+^ levels, and hormones like testosterone exemplifies the integration of biophysical and biochemical cues in the male reproductive system.

The involvement of BK channels in multiple aspects of the female reproductive system is crucial. BK channels modulate progesterone secretion in ovarian granulosa cells (GCs) (Kunz et al., 2002), and have been implicated in the modulation of intracellular K^+^ and Ca^2+^ concentrations. Blocking of BK channels with IbTX reduces progesterone secretion, indicating a potential role for these channels in regulating the release of this hormone, which is essential for the menstrual cycle and maintenance of pregnancy (Kunz et al., 2002). Furthermore, Kim et al. (2020) reported the presence of many other types of K_Ca_ channels alongside BK channels, such as intermediate conductance (IK) and small conductance (SK) channels in human GCs. The K_Ca_ channels play a role in producing sex steroid hormones that are induced by gonadotropins (Kim et al., 2020). The involvement of BK channels in intracellular calcium signaling and progesterone release in luteinizing GCs emphasizes the importance of these channels in the control of female reproductive hormones (Mayerhofer et al., 1993).

## 11 BK channel related channelopathies

Disorders associated with abnormalities in the BK channel functioning are increasingly being described. Mutations in the KCNMA1 gene are linked to neurological and neurodevelopmental illnesses. A comprehensive review of human diseases associated with KCNMA1 variants was recently published and interested readers can find it in (Meredith, 2024).

### 11.1 Epilepsy

Recurrent and unprovoked seizures due to the electrical hyperactivity of neurons are present in patients diagnosed with epilepsy (Aaronson et al., 2006). The magnitude of fAHP (fast afterhyperpolarization), which modulates AP shape is determined by BK channel currents (Latorre et al., 2017). Therefore, any alteration in BK channel activity will increase neuronal excitability. It has been suggested that epilepsy can be caused by BK channel gain-of-function (GOF) (Okawa et al., 2000; Du et al., 2005) and loss-of-function (LOF) mutations in the *KCNMA1* gene (Stafstrom and Carmant, 2015; Li et al., 2018).

The *de novo* mutation BK-D434G was discovered in patients with generalized epilepsy and paroxysmal dyskinesia (GEPD). In this is a gain-of-function (GOF) mutation, the channel opens faster, closes slower and is more sensitive to Ca^2+^ than the wild-type BK (Du et al., 2005; Ermolinsky et al., 2008). The enhancement of the allosteric coupling between the RCK1 Ca^2+^ binding site and the pore domain due to a mutation (D434G) in the S6-RCK1 linker increases the apparent Ca^2+^ sensitivity of the channel (Yang et al., 2010). To explain its pathophysiological mechanism, a knock-in mouse line carrying the D434G mutation was generated through homologous recombination. The knock-in mouse line exhibited hyperexcitability in cortical pyramidal neurons and PCs. The BK-D434G mutation increases the fAHP and reduces voltage-gated sodium (Na_v_) channel inactivation, leading to higher firing rates in cortical neurons and PCs (Yang et al., 2010).

Furthermore, the BK-N995S mutation has been reported as a GOF mutant that does not affect Ca^2+^ sensitivity. The BK-N995S mutation shifts the voltage dependence of activation even further to the left compared to the BK-D434G and wild-type channel. This results in larger AP-evoked responses (Okawa et al., 2000; Lee and Cui, 2009). The mutation was found exclusively in patients with epilepsy, suggesting a relationship between paroxysmal dyskinesia and Ca^2+^ sensitivity. Conversely, a mouse model with the BK-N995S mutation exhibited episodes of stress-induced dyskinesia (Dong et al., 2022; Ancatén-González et al., 2023). In addition, ubiquitination plays a crucial role in impeding the trafficking of polyubiquitinated BK channels from the endoplasmic reticulum (ER) to the cell membrane. Mice with a deletion of DDB1, a component of the complex E3 ubiquitin ligase CRL4A^CRBN^, exhibited augmented BK channel activity in hippocampal neurons and develop epilepsy as they age (Moldenhauer et al., 2020).

Mutation screening of patients with idiopathic generalized epilepsies (IGs) revealed a mutation in which the terminal 18 amino acids of the β3 subunit were truncated (delA750) (Lorenz et al., 2007). This deletion causes a positive shift in the voltage dependence of the BK channel, resulting in impaired repolarization. This, in turn, increases synaptic terminal excitability and neurotransmitter release (Park et al., 2022). Furthermore, rats with chronic epilepsy have exhibited a LOF in BK channels due to the downregulation of β4-subunit expression in hippocampal neurons. This finding is consistent with BK KO mouse models that display spontaneous epilepsy and motor impairment (Dong et al., 2022).

### 11.2 Fragile X syndrome

Fragile X Messenger Ribonucleoprotein (FMRP) is a protein that is widely expressed in the nervous system and regulates ion channel activity through translation, surface expression, and gating dynamics (N’Gouemo, 2011). LOF of FMRP results in Fragile X syndrome (FXS), which is characterized by intellectual disability with autistic features. These abnormalities result from changes in vesicle pool size and vesicle recycling kinetics in presynaptic terminals lacking FMRP, which alters the modulation of synaptic strength, also known as short-term plasticity (STP). STP serves multiple neuronal functions, including information processing, working memory, and decision-making (Yao et al., 2022). In terms of the cellular mechanism, FMRP interacts with the β4 subunit of the BK channel to limit AP broadening during repetitive firing. Conversely, when FMRP is deficient, BK activity decreases and AP broadening increases, resulting in higher Ca^2+^ influx, neurotransmitter release, and STP (Deng and Klyachko, 2021). For instance, FMRP-R138Q maintains RNA binding and translational regulation while eliminating BK channel modulation, and AP duration (Deng et al., 2011). Recent evidence suggests that the regulation of FMRP BK channels may be more complex. FMRP not only modulates β4, but also BK α subunit, albeit with slower gating, which may reduce of its contribution to AP repolarization (Deng et al., 2013).

### 11.3 Ataxia

BK channel dysfunction has been linked to movement disorders. Mice lacking BK channels show abnormal locomotion and ataxia. This is due to a reduction in the firing activity of PCs located in the cerebellum, which affects the olivo-cerebellar circuitry responsible for normal motricity (Myrick et al., 2015; Kshatri et al., 2020). Tremor toxins such as paxillin and loritrem B, which inhibit BK channels, do not cause locomotor deficiency in BK^−/-^ mice and reduce ataxia in β4^−/−^ mice suggesting that BK channels play a role in locomotor coordination (Chena et al., 2010). A mutation in the BK selectivity filter (G354S) was identified in a patient with progressive cerebellar ataxia, cognitive impairment, and dyskinesia (Du et al., 2020). This mutation resulted in LOF with reduced channel conductance and loss of K^+^ selectivity in plasma BK and the mitoBK channel (Sausbier et al., 2004). Additionally, BK-G354S exhibits increased Na^+^ permeability, which may alter BK function in PCs by prolonging depolarization and reducing the cytoprotective effect of mitoBK.

### 11.4 Neuropathic pain

Nociceptive information is processed in the superficial dorsal horn of the spinal cord. BK channels are expressed in small- and medium-diameter DRG neurons (Imlach et al., 2008) and play a critical role in neuropathic pain. The pathophysiology of neuropathic pain consists of increased firing of afferent terminals of excitatory synaptic transmission in the spinal cord. In rats, nerve ligation (a model of peripheral nerve injury) increases brain-derived neurotrophic factor (BDNF), which causes a reduction in both mRNA and expression of the BK channel in the DRG, leading to an increase in excitatory transmission (Scholz and Woolf, 2007; Furukawa et al., 2008). The role of spinal microglia in neuropathic pain involves the β3 subunit, as *KCNMB3* ^−/−^ mice suggested. These mice show a reduction in BDNF and inflammatory molecules, which attenuates neuropathic pain (Chen et al., 2009; Cao et al., 2012). The N-terminus of the BK channel inhibits the Ca_v_α2δ subunit, which oversees Ca_v_ trafficking to presynaptic afferent terminals in the DRG and increases neurotransmitter release suggesting that the BK channel acts as a “brake” on neuronal hyperexcitability (Hayashi et al., 2016). Another proposed mechanism is ER stress, which results from prolonged inflammation that can disrupt protein folding. These conditions can change ER and cytosolic Ca^2+^ dynamics, which may cause a positive shift in BK channel voltage dependence and lead to hyperexcitability in the DRGs (Zhang et al., 2018; Imari et al., 2019).

Epigenetic regulation plays an essential role in nociceptive transmission. Nerve injury increases histone-lysine N-methyltransferase-2 (G9a) activity, which silences not only the *KCNMA1* gene but also the *KCNA4* (K_v_1.4), *KCND2* (K_v_4.2) and *KCNQ2* (K_v_7.2) genes through histone H3 demethylation in DRG neurons (Smith, 2020).

A hypothesis links the BK channel to the endocannabinoid system. The CB1 receptor is expressed in nerve terminals in the periphery, and its activation has an analgesic effect (Dopico et al., 1996; Li et al., 2019). Recent evidence from mice with chronic constriction injury (CCI)-induced neuropathic pain suggests that activation of CB1 receptors can suppress increased firing and induce analgesia. However, when IbTX was applied, these effects were abolished, indicating that BK activity is important for the CB1-induced analgesic effect (Li et al., 2019).

### 11.5 Ethanol sensitivity

Ethanol (EtOH) activates BK channels at neurohypophysial terminals and modulates vasopressin and oxytocin release. However, at the cell body and dendrites, EtOH does not affect BK channels (Li et al., 2019). Furthermore, EtOH induces vasoconstriction in cerebral artery myocytes by inhibiting BK(α+β1) channel STOCs (Dopico et al., 1996). The diverse effects of β subunits on EtOH-induced potentiation of BK currents are because β1 deficiency accelerated the escalation of EtOH drinking during withdrawal under conditions of chronic intermittent ethanol (CIE) in mice, whereas β4 absence attenuated it (Wynne et al., 2009). This is consistent with the differential expression of the β1 subunit in the soma and the β4 subunit in nerve terminals (Li et al., 2019). It is interesting to note that the EtOH binding site is reported to be close to the RCK1 site. Furthermore, substitutions at residues T352 and K361 eliminate EtOH binding and the associated behavioral response without affecting BK conducting properties (Bukiya et al., 2009; Kreifeldt et al., 2013). Additionally, EtOH may modify BK channel trafficking to the cell surface, leading to increased BK channel internalization after 3–6 h, which has been proposed to be the basis of alcohol tolerance (Bukiya et al., 2014; Contet et al., 2016).

It is unclear whether the interaction of EtOH with BK channels directly contributes to intoxication or chronic tolerance alcohol escalation (Imlach et al., 2008). Both hypotheses are supported by behavioral evidence. For example, in *C. elegans*, alcohol intoxication depends on BK channel ortholog (SLO-1) clusters (Davis et al., 2014) at presynaptic terminals and in the sarcolemma, which couple to Ca^2+^ channels that mediate behavioral sensitivity to ethanol (Berkefeld et al., 2006; Palacio et al., 2015).

### 11.6 Hypertension

Hypertension is characterized by increased arterial tone due to the elevation of global Ca^2+^ in VSM. The BK channel modulates STOCs, inhibiting Ca_v_ channels, acting as an “emergency brake” and negative feedback for muscle contraction (Latorre et al., 2017). This is mainly because the β1 subunit increases BK channel Ca^2+^ spark sensitivity. β1 KO rats have shown a reduction in STOCs and, in turn, promotion of vasoconstriction (Bettinger and Davies, 2014; Oh and Kim, 2019). Additionally, the frequency of STOCs is reduced in SMCs from hypertensive patients. The frequency of measuring STOCs in VSMCs was found to be reduced in hypertensive patients. Furthermore, mesenteric arterial tissue showed a reduction in β1 mRNA, but not in the BK α subunit (Amberg et al., 2003; Nieves-Cintrón et al., 2007). On the other hand, a GOF mutant of the β1 subunit (E65K) is associated with a low prevalence of moderate and severe diastolic hypertension (Amberg and Santana, 2003; Gonzalez-Perez et al., 2022), suggesting a cardioprotective role for this polymorphism (Yang et al., 2013).

Hypertensive patients often have many agonistic autoantibodies against the AT1R (AT1-AA) in their serum (Fernández-Fernández et al., 2004). The AT1R can inhibit BK channel activity by forming a protein-protein complex in renal arterial myocytes’ cell periphery and downregulating it through PKC phosphorylation. (Sentí et al., 2005; Gonzalez-Perez et al., 2022). Recently, the therapeutic effect of the BK channel has been tested in AT1-AAs-positive rats. NS1619 increased the lumen, remodeled vascular tone, and improved resistance in mesenteric arteries (Xin et al., 2018).

### 11.7 Erectile dysfunction

Erectile dysfunction (ED) is a condition where a sustained penile erection cannot be maintained due to a reduction in blood supply to the corpora cavernosa. This results in penile tumescence due to the inability of the corpus cavernosum smooth muscle (CCSM) to relax, favoring its contracted state and flaccidity (Zhang Z. et al., 2014). ED can be caused by a reduction in nitric oxide that decreases cGMP and its modulation of several kinases that activate various K^+^ channels. The relevance to ED of the BK channel has been extensively described using pharmacological and BK KO studies in which LOF abolishes CCSM relaxation (Wang, 2008; Grimm et al., 2009; Gareri et al., 2014).

The pharmacological treatment of ED primarily involves using phosphodiesterase type 5 inhibitors, such as sildenafil. However, their effectiveness may not extend to all patients, particularly those with risk factors such as diabetes (Diniz et al., 2020). Therefore, BK channels could serve as a viable therapeutic alternative. Several BK channel activators have been tested for this purpose. LDD175 was tested in diabetic rats and demonstrated a reduction in diabetes parameters and an improvement in penile erection. Additionally, it had an additive effect when combined with sildenafil (Spektor et al., 2002). In contrast, NS11021 increases CCSM BK currents *in vitro* and facilitates the erectile response in anesthetized rats (Hatzimouratidis et al., 2016).

### 11.8 Overactive bladder

Overactive bladder (OAB) and urinary incontinence occur when the excitability of bladder smooth muscle (BSM) cells increases (Figure 3). The mechanism behind this remains to be determined. However, evidence suggests that the BK channel plays a role in β-adrenergic receptor-induced BSM relaxation (Sung et al., 2017). Additionally, LOF increases BSM contractility and urinary frequency without affecting Ca^2+^-sparks or K_v_ currents (Kun et al., 2009). This statement is in line the finding in rats with OAB induced by diabetes, which demonstrates a decrease in *KCNMA1* and *KCNMB1* mRNA levels in BSM (Andersson et al., 2021).

## 12 Concluding remarks

This review explores the roles of BK channels in cellular physiology and diseases resulting from their abnormal functioning. BK channels modulate membrane potential, regulated neurotransmitter release, and maintain vascular tone, making them versatile regulators that orchestrate cellular responses with precision. The wide range of tissues that express BK channels highlights their essential role in maintaining homeostasis and adapting to various physiological demands.

The biophysics of BK channels reveals an interplay between voltage, Ca^2+^, and auxiliary subunits that provide functional diversity, regulating channel openings and closures. The synergy between structural elements and dynamic interactions provides a nuanced understanding of the channel’s behavior, offering a base for future investigations into channelopathies and therapeutic interventions.

The identification of BK channel mutations linked to various pathologies has elevated the channel’s clinical relevance. Understanding the mechanistic underpinnings of these mutations not only provides insights into disease etiology but also offers potential diagnostic markers and therapeutic targets for a spectrum of disorders. The realization that BK channels can be targeted by drugs has opened up exciting therapeutic possibilities, from modulating vascular tone to alleviating neuronal excitability. The pharmacological manipulation of BK channels holds promise for treating a diverse range of conditions.
